# Age-related dopaminergic integrity in the SNpc and VTA: morphometric and volumetric findings in marmoset (*Callithrix jacchus*)

**DOI:** 10.3389/fnana.2026.1768392

**Published:** 2026-03-10

**Authors:** Maria M. O. Azambuja, Nelyane N. M. de Santana, Paulo L. A. G. Morais, Gabriel A. M. Vasiljevic, Jeferson S. Cavalcante, Adhil Bhagwandin, José R. L. P. Cavalcanti, Rovena Clara Engelberth

**Affiliations:** 1 Laboratory of Neurochemical Studies, Department of Physiology and Behavior, Bioscience Center, Federal University of Rio Grande do Norte, Natal, Brazil; 2 Laboratory of Experimental Neurology, College of the Health Sciences, State University of Rio Grande do Norte, Mossoró, Brazil; 3 Edmond and Lily Safra International Institute of Neuroscience, Santos Dumont Institute, Macaíba, Brazil; 4 Division of Clinical Anatomy and Biological Anthropology Department of Human Biology, Faculty of Health Sciences University of Cape Town, Cape Town, South Africa

**Keywords:** aging, dopamin, marmoset, substantia nigra, tyrosine hydroxylase, ventral tegmental area

## Abstract

Given a convergence of evidence indicating age-related vulnerability in nuclei associated with basal ganglia circuits, understanding the pattern of normal aging in non-human primates is essential for basic and applied research. To address this, we examined the age-dependent vulnerability of dopaminergic cells in the substantia nigra pars compacta (SNpc) and ventral tegmental area (VTA) of marmoset through morphoquantitative analysis of cytoarchitecture. Thus, we selected brain tissue from adult and aged marmosets processed for tyrosine hydroxylase (TH) immunohistochemistry. We estimated regional volume and counted TH-immunopositive (TH^+^) neurons in the SNpc and VTA. Statistical comparisons used permutation tests and Spearman’s tests to analyze differences between age groups. Although Spearman’s correlation test showed a negative correlation between regional volume and age and between TH^+^ cell number and age, no significant differences were found in either the permutation test or Spearman’s correlation for TH + neuronal number and for regional volume between the age groups for both nuclei. We concluded that aging in marmosets does not lead to significant loss of dopaminergic neurons or measurable volumetric reduction in the SNpc or VTA. Our results highlight the importance of understanding physiological aging in contrast to models characterized by structural degeneration, such as those found in pathological conditions. Understanding, in a promising experimental model as marmoset, the pattern of vulnerability and resilience of dopaminergic regions fills gaps in the literature and opens avenues for understanding molecular and functional changes related to aging.

## 1 Introduction

Aging is a complex and multifactorial process, marked by a series of modifications in biological systems over chronological time (Medawar, 1952; López-Otín et al., 2013, 2023). It unfolds differentially at the tissue, cellular, and molecular levels, and its intrinsic mechanisms have the potential to render specific regions of the nervous system more vulnerable to functional decline, particularly cognitive functions (Cochar-Soares et al., 2021; Pandya and Patani, 2021; Sala et al., 2021).

Over the last decades, numerous studies have sought to elucidate how aging affects the nervous system. In this context, changes have been described ranging from macroscopic features, such as reduced brain size and enlarged cortical sulci, to microscopic alterations involving decreased neuronal numbers and synaptic density in specific regions (Dekaban and Sadowsky, 1978; Barnes, 2003). In addition, important cellular and molecular modifications have been identified, including alterations in G protein-coupled receptor signaling (Kelly, 2018) and increased production of inflammatory cytokines (Lin et al., 2018).

Among the various neurotransmitter-mediated circuits in the central nervous system, the dopaminergic system is perhaps one of the most extensively studied. Throughout the central nervous system (extending even to the retina), 10 nuclear groupings express dopamine as their principal neurotransmitter (see further details in Björklund and Dunnett, 2007). Of these, three nuclei are located in the midbrain and are known as the substantia nigra pars compacta (SNpc), the ventral tegmental area (VTA), and the retrorubral field. It is important to highlight that more than 70% of all dopamine found in the central nervous system is related solely to the SNpc and the VTA (German et al., 1983; Pakkenberg et al., 1991), and it plays a pivotal role in mechanisms such as motor control, motivation, and reward, and is also implicated in highly prevalent neuropsychiatric conditions, including Parkinson’s disease, schizophrenia, and addiction (Björklund and Dunnett, 2007), which make these structures the focus of numerous studies. Although considerations regarding aging in certain dopaminergic nuclei remain scarce (Rocha et al., 2024), converging evidence indicates age-related vulnerability in nuclei associated with basal ganglia circuits, such as the SNpc (Eggers et al., 2025; Fan et al., 2022; Giguère et al., 2018) and the VTA (Lebowitz and Khoshbouei, 2020).

Cavalcanti et al. (2016) provided a detailed description of the cytoarchitecture of these nuclei in young common marmosets (*Callithrix jacchus*), enabling the SNpc and VTA to be more thoroughly explored in experimental models using this species as a reference. The marmoset is a New World primate endemic to Brazil that, in addition to its notable evolutionary proximity to humans, exhibits behavioral, physiological, and anatomical characteristics that support robust translational inferences. Regarding motor behavior, the species shows a “head-to-tail” developmental trajectory similar to that observed in humans (Wang et al., 2014) and demonstrates advanced planning of motor sequences (Matsuzaki and Ebina, 2020). Moreover, age-dependent characteristics can be identified from approximately 6 years of age, with the elderly stage commonly considered established around 8 years (Tardif et al., 2011; Abbott et al., 2003).

In this context, given the functional relevance of the SNpc and VTA, understanding their patterns of selective vulnerability extends beyond basic research and reaches applied domains, with potential implications for quality of life and the treatment of neurodegenerative diseases. Thus, this study aimed to describe the morphoquantitative aspects of the cytoarchitecture of dopaminergic cells in the SNpc and VTA across the normal aging process of the marmoset, providing an essential overview to address the current scarcity of data regarding the integrity of dopaminergic systems in non-human primates.

## 2 Materials and methods

Serial brain sections of five marmosets (three aged and two adults; 235–289 g) obtained from the Primatology Center of the Federal University of Rio Grande do Norte were used in the present study (see Table 1 for details). These animals had also been included in previous chemoarchitectonic investigations conducted in our laboratory (Engelberth et al., 2013; Cavalcanti et al., 2016; Santana et al., 2025), which provide a comprehensive description of tissue processing and immunohistochemical protocols. Sex was not adopted as an exclusion criterion. All procedures complied with Brazilian legislation (Law No. 11.794/2008) governing the ethical use of animal research and were approved by the institutional ethics committee (CEUA-UFRN, protocols 026/2010 and 014/2014). All animals were clinically healthy and exhibited no behavioral abnormalities or stereotypies suggestive of neurological dysfunction. Marmosets were identified as aged from 96 months old (Kramer and Burns, 2019).

**Table 1:** Case overview.

Case#	Age (months)	Sex	SNpc-TH^+^cell/mm^2^	VTA-TH^+^ cell/mm^2^	Volume SNpc (mm^3^)	Volume VTA (mm^3^)
Sagui	48	M	4,140	2,359	0.86	1.62
SJ1	39	F	4,581	2,739	0.71	1.72
SI4	143	M	2,967	2,024	0.60	1.24
SI5	123	M	2,985	1,592	0.62	1.31
SI6	121	F	3,475	1,890	0.71	1.49

All animals were pre-medicated with xylazine (0.5 mg/kg) and tramadol hydrochloride (5 mg/kg), and then deeply anesthetized with 5% isoflurane delivered in 100% oxygen as the carrier gas. Subsequently, intracardiac perfusion was performed using heparinized saline, followed by 4% paraformaldehyde in 0.1 M phosphate buffer (PB, pH 7.4). The dissected brains were post-fixed in the same medium for 24 h and cryoprotected in a 30% sucrose solution for 1–2 days. Brains were sectioned at 30 μm on a freezing microtome (SM2000 R, Leica Biosystems, Nussloch, Germany). Every sixth section was collected for TH immunostaining and storage in anti-freezing solution at −20 °C.

Details of the immunohistological procedures have been described previously (Cavalcanti et al., 2016). Briefly, representative sections of SNpc and VTA were incubated overnight with a monoclonal mouse anti-TH primary antibody (Sigma-Aldrich, #Cat T1299, RRID: AB_477560) in a 1:10,000 dilution containing BSA and 0.1 M PB with 0.4%Triton X-100 (PBTX). After rinsing, sections were incubated with a donkey anti-mouse secondary antibody (Jackson ImmunoResearch, Cat# 715–001-003, RRID: AB_2340755) in a 1:1,000 dilution with PBTX for 90 min. Then, sections were incubated with avidin-biotin-HRP complex (Vectastain standard ABC kit, PK-6100, Vector Laboratories) for 90 min. TH-immunopositive (TH^**+**^) elements were then visualized with a 3,3′-diaminobenzidine (DAB) solution combined with hydrogen peroxide. Stained tissue was dehydrated in a graded ethanol series, cleared, and coverslipped using Entellan mounting medium (Merck, Darmstadt, GER).

Photomicrographs at 2.5x, 5x, and 20x objective lens from the SNpc (interaural +6.00 mm to +3.00 mm) and VTA (interaural +6.00 mm to +5.30 mm) were acquired with a digital video camera (CX 9000, MBF Bioscience, Williston, United States) connected to Axio Imager Z2 optical microscope (Zeiss, Oberkochen, Germany). Using the software Canvas 12 (ACD Systems, Victoria, Canada, RRID: SCR_014288), all selected images were minimally processed for brightness and contrast and subsequently employed to generate the figures and illustrations. The identification of the SNpc and VTA was based on chemoarchitectonic criteria previously described (Cavalcanti et al., 2016) and aligned with the marmoset brain atlas (Paxinos et al., 2012).

**Figure 1:**
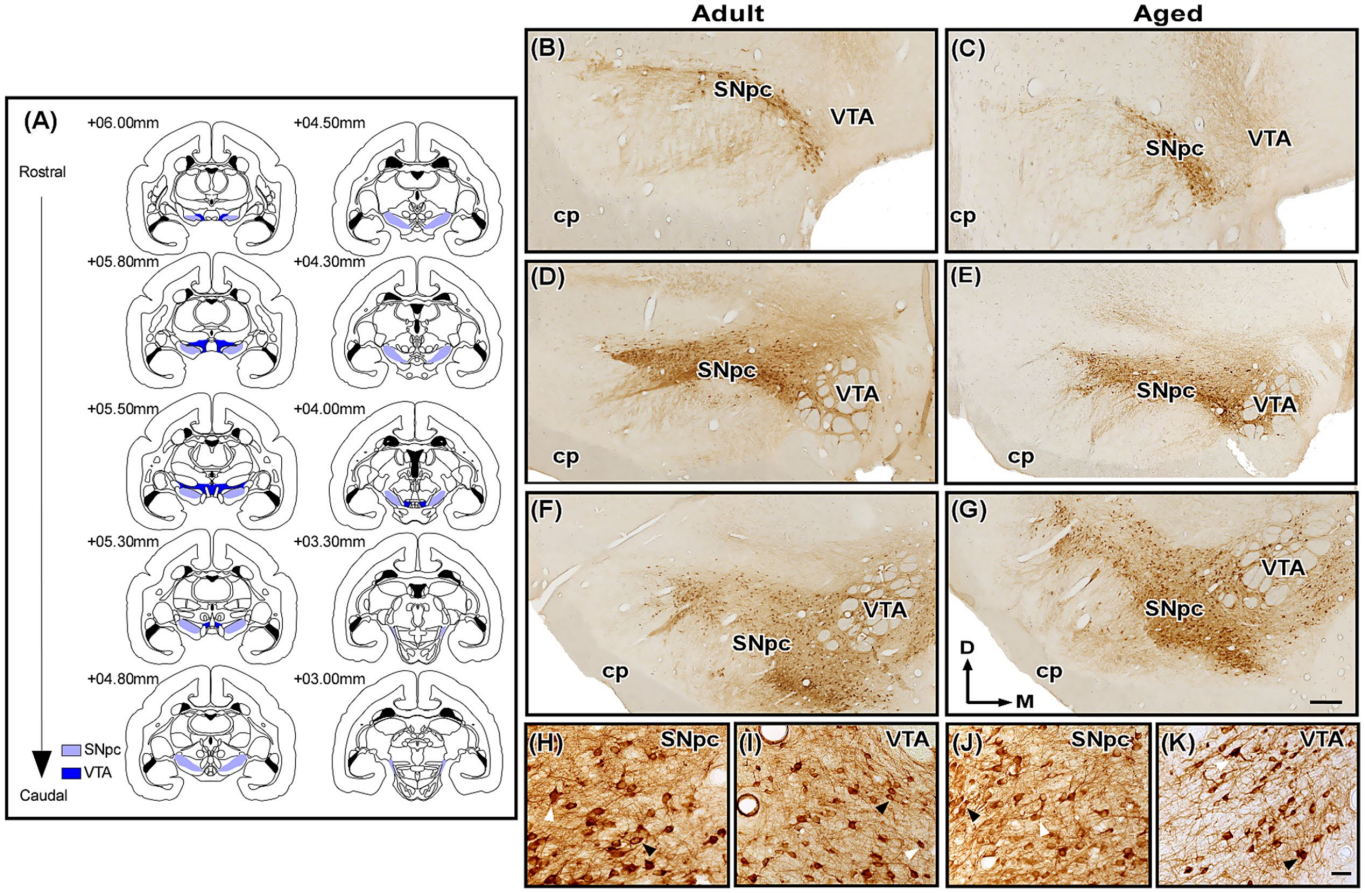
Photomicrographs of TH-immunostained histological sections containing the marmoset substantia nigra pars compacta (SNpc) and ventral tegmental area (VTA). **(A)** Representative sections of the SNpc and VTA used in the present analysis. **(B–G)** TH immunoreactivity in the SNpc and VTA of adult (left) and aged (right) animals at rostral **(B,C)**, middle **(D,E)** and caudal **(F,G)** levels (~ + 6.00 mm to +4.00 mm). **(H,I)** High magnification photomicrographs of TH-immunolabeled SNpc and VTA neurons of adult animals. **(J,K)** High magnification photomicrographs of TH-immunolabeled SNpc and VTA neurons of aged animals. Black arrowheads point to neurons with an evident nucleus, while white arrowheads indicate cells excluded from the counting due to o the presence of a nucleus not identifiable. Scale bar: 500 μm **(B–G)** and 50 μm **(H–K)**. cp, cerebral peduncle. Adapted from Paxinos et al. (2012).

To assess regional volume, representative sections of SNpc and VTA (Figure 1A) were analyzed bilaterally in each animal at 2.5 × objective lens using the trapezoidal principle, a morphometric estimation based on systematically sampled serial sections (Rosen and Harry, 1990). This measurement was conducted with the ImageJ 1.54v software (NIH, Bethesda, United States, RRID: SCR_003070) and the final volume was calculated using an algebraic formula described by Díaz et al., 2003:*V* = (*S*_1_ *+ S_2_*) *× d/2 +* (*S_2_ + S_3_*) *× d/2 +* …(*S_n-1_ + S_n_*) *× d/2*

Where *d* represents the interval between consecutive sections, and *S_1_…S_n_* corresponds to the cross-sectional area of the region of interest in each analyzed section. For the analysis of neuronal number, TH^+^ neuronal profiles with a clearly visible nucleus (Figures 1H–K) were bilaterally quantified in the SNpc and VTA, with sections spaced 180 μm apart and examined using low-magnification images (5×). All sections were blind-coded, and TH + neurons were counted manually using ImageJ. Importantly, a 5 × magnification was selected for this morphometric analysis due to the relatively large size of TH^+^ neurons in both nuclei in the marmoset, which allowed for the visualization and discrimination of individual neuronal profiles in both adult (Figures 1H,I) and aged (Figures 1J,K) groups, while maintaining a broad field of view. For both morphometric analyses, 9 SNpc and VTA sections were selected from a total of 17 and 15 SNpc and VTA sections, respectively.

A Shapiro–Wilk test was conducted to evaluate the data normality. Differences between age groups were examined via permutation tests, using 100,000 sub-sample interactions (see Table 2 for confidence intervals). These statistical procedures were performed in Python version 3.10, and data are expressed as mean ± standard deviation. Correlations between age and assessed variables were analyzed using Spearman’s test in GraphPad Prism version 10.0 (GraphPad, San Diego, United States, RRID: SCR_002798). Statistical significance was set at *p* < 0.05.

**Table 2:** Mean values, observed difference for each factor in the regions of interest, and confidence intervals (95%).

Region	Factor	Mean ± SD (adult)	Mean ± SD (aged)	Observed mean difference (adult—aged)	Lower bound	Upper bound
SNpc	TH^+^ neuronal number	4,360.5 ± 311.83	3,142.33 ± 288.33	1,218.17	129.025	2,436.525
	Volume	0.78 ± 0.11	0.64 ± 0.06	0.14	−0.008	0.283
VTA	TH^+^ neuronal number	2,549 ± 268.7	1,835.33 ± 221.12	713.67	81.644	1,428.311
	Volume	0.167 ± 0.07	1.35 ± 0.13	0.32	−0.012	0.646

## 3 Results

Immunolabeling for TH was robust, with TH^**+**^ elements distributed throughout the anteroposterior extent of both the SNpc and VTA (Figures 1B–G). This staining revealed a higher prevalence of pyramidal and multipolar neurons in the area equivalent to the SNpc, whereas fusiform and bipolar-appearing neurons were more frequently observed along the VTA. In both nuclei, dendritic organization patterns could not be clearly distinguished due to the diffuse distribution of dendritic processes (Figures 1H–K). Qualitatively, the adult group exhibits more intense TH^**+**^ staining compared to the aged group in equivalent stereotaxically levels (Figures 1B–G).

**Figure 2:**
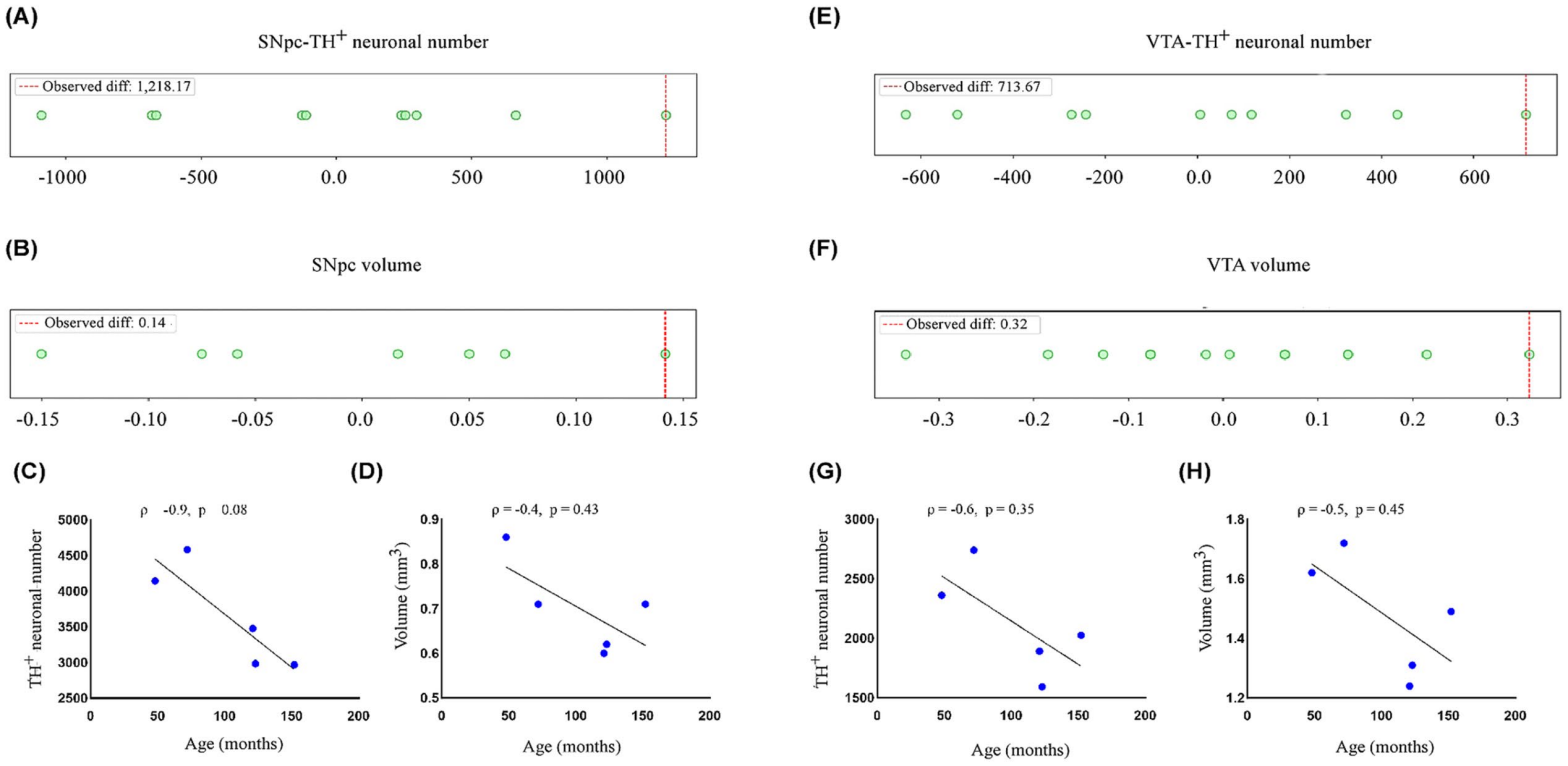
Morpho-quantitative parameters in marmoset substantia nigra pars compacta (SNpc) and ventral tegmental area (VTA). **(A,B)** Dot plot illustrates the distribution and statistical comparison between adult (*n* = 2) and aged (*n* = 3) animals for **(A)** TH^+^ neuronal number and **(B)** regional volume (in mm^3^) in the SNpc. The permutation test revealed no significant difference for TH^+^ neuronal count (*p* = 0.10165) and regional volume (*p* = 0.16503). These findings were supported by Spearman correlation tests, which indicated **(C)** TH^+^ neuronal number (*ρ* = −0.9, *p* = 0.08) and **(D)** regional volume (ρ = −0.4, *p* = 0.43) do not vary significantly with age. **(E,F)** Dot plot illustrates the distribution and statistical comparison of age groups for **(E)** TH^+^ neuronal number and **(F)** regional volume (in mm^3^) in VTA. The permutation test showed no significant difference between age groups for TH^+^ neuronal count (*p* = 0.09947) and regional volume (*p* = 0.19798). Correlation analyses further support these findings, suggesting a no linear correlation between age and **(G)** TH^+^ neuronal number (ρ = −0.6, *p* = 0.35) and **(H)** regional volume (ρ = −0.5, *p* = 0.45). For the permutation test, each point represents a mean difference obtained from a permuted dataset. For correlation plots, each point represents one individual. Significance at *p* > 0.05.

The number of TH^**+**^ neurons in the SNpc and VTA (Table 1) was obtained using the morphometric analysis of cellular profiles. As shown in Figures 2A,E, the permutation test revealed no significant difference for TH^**+**^ neuronal number between age groups for both nuclei (SNpc, observed mean difference = 1,218.16, permuted *p* = 0.10165, observed Cohen’s *d* = 4.11; VTA, observed mean difference = 713.6, permuted *p* = 0.09947, observed Cohen’s *d* = 2.99). These findings support the qualitative observation that dopaminergic cell numbers in both the SNpc and VTA do not differ between adult and aged animals. Similarly, the Spearman’s correlation analysis demonstrated a negative correlation between TH^**+**^ cell number and age for both nuclei (SNpc, Spearman rank correlation coefficient *ρ* = −0.9, *p* = 0.08; VTA, ρ = −0.6, *p* = 0.35), although they were not statistically significant (Figures 2C,G).

The volumetric analysis of SNpc and VTA (Table 1) was performed using the trapezoidal principle (Rosen and Harry, 1990). Qualitatively, no differences in regional volume were observed between adult and aged animals (Figures 1B–G). Indeed, this observation was corroborated by permutation test, which demonstrated no significant difference in mean regional volume between age groups (SNpc, observed mean difference = 0.14166, permuted *p* = 0.16503, observed Cohen’s *d* = 1.82; VTA, observed mean difference = 0.3233, permuted *p* = 0.19798, observed Cohen’s *d* = 2.86) (Figures 2B,F). Additionally, the Spearman correlation test showed a negative, albeit not significant, correlation between regional volume and age for both nuclei (SNpc, Spearman rank correlation coefficient ρ = −0.4, *p* = 0.43; VTA, ρ = −0.5, *p* = 0.45) (Figures 2D,H).

## 4 Discussion

In the present study, we examined whether aging impacts dopaminergic integrity in the SNpc and VTA of adult and aged marmosets through morphoquantitative analysis of TH^+^ neurons and regional volumetry. We found no statistically significant differences between age groups, although a modest age-related negative trend in TH expression was observed in both nuclei. These findings align with previous studies reporting that aging, while associated with functional dopaminergic decline, does not necessarily result in substantial neuronal loss in the SNpc or VTA, including evidence from non-human primates (Barata-Antunes et al., 2020; Collier et al., 2007; Emborg et al., 1998).

A considerable body of classical and contemporary evidence indicates that aging-related dopaminergic alterations are often driven by molecular, metabolic, and synaptic changes, such as reduced dopamine levels, decreased D2 receptor density, changes in axonal homeostasis, and impaired mitochondrial function, without detectable neuronal death at the morphological level (Karrer et al., 2017). This dissociation between structure and function is consistent with our results: the maintenance of TH^+^ neuronal number in the SNpc and VTA, despite qualitatively reduced immunoreactivity, may reflect transcriptional, metabolic, or regulatory alterations rather than overt neuronal loss. Our data are consistent with other quantitative studies of the SNpc in elderly rhesus monkeys (Siddiqi and Peters, 1999) and humans (Di Lorenzo Alho et al., 2016), which generally report a trend of volume loss in the SNpc and a reduction in dopaminergic neurons. This structure–function dissociation aligns with our findings: the preserved number of TH^+^ neurons in the SNpc and VTA, despite their qualitatively reduced immunoreactivity, may reflect altered transcriptional and metabolic regulation rather than substantial neuronal loss. This observed preservation is also consistent with reports indicating that the SNpc and VTA display differential vulnerability during aging (Flores-Ponce and Velasco, 2024). The absence of significant neuronal loss observed here also aligns with emerging evidence that the SNpc and VTA exhibit distinct vulnerabilities during aging. Recent work has highlighted bioenergetic, structural, and transcriptional differences that render VTA neurons relatively less susceptible to age-related dopaminergic degeneration compared to SNpc neurons (Phan et al., 2025). These intrinsic distinctions help explain why neurodegenerative diseases such as Parkinson’s disease disproportionately affect the SNpc while largely sparing the VTA.

Within this framework, two recurring themes in the aging literature become relevant: (1) the heterogeneous vulnerability of dopaminergic subregions, and (2) the presence of compensatory mechanisms in certain species or nuclei that preserve dopaminergic function despite cellular stress. Regarding neuronal vulnerability, Prakash (2022) and Ni and Ernst (2022) emphasize that the SNpc, particularly its ventrolateral subdivision, exhibits time-dependent vulnerability driven by high energetic demand, oxidative stress, and dopamine-related metabolic load, which together contribute to cellular dysfunction. However, other studies report differing susceptibilities depending on species or cellular markers. For example, in mice, Barata-Antunes et al. (2020) found no evidence of age-related loss of TH-immunoreactive neurons, though the region exhibited marked microglial activation, possibly reflecting an early stage preceding neuronal loss (Mirarchi et al., 2024). Although the VTA is generally considered more resilient than the SNpc, this resilience is not absolute. Reports in non-human primates describe neuronal loss and dopaminergic decline in specific VTA subregions, such as the paranigral and parabrachial pigmented nuclei (Emborg et al., 1998; Siddiqi et al., 1999). These findings underscore the importance of rigorously examining aging effects across species and subregions.

Turning specifically to the volumetric findings, our analysis revealed no significant differences in the regional volume of the SNpc or VTA between adult and aged animals. Although a slight negative association between age and volume was observed, it did not reach statistical significance. This pattern is consistent with studies reporting that gross anatomical measures of dopaminergic nuclei often remain stable during normal aging, particularly in rodents and non-human primates (Collier et al., 2011; Cruz-Muros et al., 2006; Hardman et al., 2002). Age-related dopaminergic decline frequently manifests through biochemical, electrophysiological, or synaptic alterations before structural atrophy becomes detectable. Moreover, rat, human, and primate studies increasingly suggest that early aging-related dopaminergic decline appears in functional and neuromelanin-based MRI markers rather than in regional volume *per se* (Pérot et al., 2025; Watanabe, 2024).

From a methodological perspective, the principal limitation of our study is the number of animals, reflecting the inherent difficulty in obtaining aged marmosets (≥96 months) (Kramer and Burns, 2019). Most experimental marmoset colonies, including our primatology center, have a median lifespan of 60–72 months (Ross, 2019), making individuals older than 129 months exceptionally scarce. The number of adult animals was further constrained by restricting analyses to well-characterized specimens processed using identical histological and immunohistochemical protocols, a methodological choice adopted to minimize experimental variability, as these animals had been previously used in other studies from our laboratory (Engelberth et al., 2013; Cavalcanti et al., 2016; Santana et al., 2025). However, the use of permutation tests, as applied here, is particularly appropriate for small samples typical of primate research. Such approaches are recognized as statistically robust even under reduced sample sizes and non-parametric distributions (Noguchi et al., 2021; Horiguchi and Uno, 2020). Therefore, the absence of significant differences in neuronal counts and volumetry should be interpreted as reliable evidence of structural preservation, rather than as a false-negative result due to sample size constraints. Future studies with larger and sex-balanced samples will be necessary to further investigate age-related changes in dopaminergic territories.

In summary, our findings indicate that aging in marmosets does not necessarily lead to a significant loss of dopaminergic neurons or measurable volumetric reduction in the SNpc or VTA. These results support models proposing that robust structural degeneration is characteristic of pathological condition**s**, such as Parkinson’s disease, rather than of physiological aging. Our data contribute to comparative neurobiology by emphasizing the need to distinguish molecular and functional aging-related changes from anatomical alterations when investigating dopaminergic systems in primates.

## Data Availability

The original contributions presented in the study are included in the article/supplementary material, further inquiries can be directed to the corresponding author.
